# Optimal dose of aerobic exercise for improving postpartum depression, anxiety, and quality of life: a meta-analysis of randomized controlled trials and dose–response analysis

**DOI:** 10.3389/fpubh.2026.1807903

**Published:** 2026-04-21

**Authors:** Luqiong Cai, Yuqing Du, Yihui Huang, Renjun Yang

**Affiliations:** 1 Department of Rehabilitation, Ningbo Rehabilitation Hospital, Ningbo, China; 2 Ningbo Psychiatric Hospital, Ningbo, China; 3 Department of Rehabilitation, Jintang First People’s Hospital, Chengdu, China

**Keywords:** aerobic exercise, dose–response analysis, postpartum anxiety, postpartum depression, quality of life

## Abstract

**Background:**

Postpartum depression, anxiety, and reduced health related quality of life (HRQoL) are common in the first year after childbirth, a period characterized by marked hormonal fluctuations, sleep disruption, and psychosocial role transitions that increase vulnerability to emotional distress, and have substantial consequences for maternal and child well-being. Aerobic exercise is increasingly recommended as a low-risk behavioral strategy, as it has been shown to improve mood, reduce stress, and modulate neurobiological pathways associated with depression, yet its effectiveness and optimal prescription parameters for postpartum mental health remain unclear. This study evaluated the effects of aerobic exercise on depressive symptoms, anxiety, and HRQoL, and characterized corresponding dose response patterns.

**Methods:**

PubMed, Embase, CENTRAL, Web of Science, and Google Scholar were searched from inception to 31 October 2025. Randomized controlled trials lasting at least four weeks and comparing structured aerobic exercise with inactive or minimal control conditions were included. Outcomes were depressive symptoms, anxiety, and HRQoL assessed using validated instruments. Random effects meta-analyses generated standardized mean differences (SMDs). Dose response relationships were examined using model-based methods.

**Results:**

Seventeen trials involving 2,865 women met inclusion criteria. Aerobic exercise significantly reduced depressive symptoms (SMD − 0.37, 95% CI − 0.60 to −0.14), with larger improvements in women with baseline depression and in interventions delivered postpartum. Dose response analysis showed a U-shaped pattern, with maximal benefit at approximately 570 MET minutes per week. No significant effect was found for anxiety (SMD − 0.11, 95% CI − 0.31 to 0.09). Aerobic exercise improved HRQoL (SMD 0.45, 95% CI 0.29 to 0.60), and an inverted U-shaped dose response indicated optimal gains near 420 MET minutes per week.

**Conclusion:**

Aerobic exercise reduces postpartum depressive symptoms and improves HRQoL. Optimal effects were observed at approximately 570 MET minutes per week for depression and 420 MET minutes per week for HRQoL, suggesting that a weekly volume of 400 to 600 MET minutes may be a practical target in postpartum care. Evidence remains insufficient to confirm anxiolytic effects.

## 1 Introduction

Postpartum mental health refers to a spectrum of emotional and functional outcomes during the first year after childbirth, including depressive symptoms, anxiety, and health related quality of life. Postpartum depression is characterized by persistent low mood, loss of interest, guilt, and impaired functioning, while postpartum anxiety often presents as excessive worry, tension, and somatic arousal that interferes with daily care (1). Current epidemiological data suggest that approximately 10 to 20 percent of women experience clinically significant postpartum depression, and similar proportions report elevated anxiety symptoms and reduced quality of life (2). This increased vulnerability may reflect postpartum hormonal changes, sleep disruption, physical recovery demands, and psychosocial stressors related to caregiving burden and barriers to help-seeking (1, 3). With global birth rates, this translates into millions of affected women each year, particularly in low- and middle-income settings. These conditions are associated with disrupted mother–infant bonding, breastfeeding difficulties, poorer physical recovery, and an increased risk of recurrent mood disorders. They also predict adverse emotional and cognitive development in offspring and generate substantial healthcare costs, productivity loss, and caregiving burden for families and society (4, 5). These findings highlight the need to prioritize prevention and treatment strategies targeting postpartum depression, anxiety, and quality of life.

Pharmacological and psychological therapies are the most frequently used interventions for postpartum mental health problems. Antidepressant and anxiolytic medications can reduce symptoms, but their use during pregnancy and lactation raises concerns about infant exposure, side effects, and long-term medication dependence (6). Access to evidence-based psychotherapies such as cognitive behavioral therapy or interpersonal therapy is often limited by financial cost, travel, childcare demands, and the availability of trained professionals (3). Many women are reluctant to seek formal mental health care because of stigma or fear of being perceived as inadequate mothers. As a result, a substantial proportion of women remain untreated or undertreated. These limitations create a clear rationale for exploring safe, acceptable, and scalable behavioral strategies. Structured physical activity and exercise are attractive in this context, as they can be integrated into daily life, support physical recovery and weight management, and confer psychological benefits such as improved mood, self-efficacy, and sleep quality (7, 8). These benefits may relate to improved stress regulation and endorphin-mediated mood effects, as well as higher self-esteem and reduced rumination (9).

Accumulating evidence suggests that exercise is a promising intervention for postpartum mental health. Randomized controlled trials indicate that structured physical activity can reduce depressive and anxiety symptoms and improve health related quality of life in perinatal populations. Prior reviews and meta-analyses have reported beneficial effects of exercise and have suggested that aerobic modalities such as brisk walking, jogging, or cycling may be particularly effective for depressive symptoms and quality of life (10, 11).

Despite growing evidence that exercise can benefit postpartum mental health, the specific contribution of aerobic exercise, the influence of key prescription characteristics, and the shape of the dose response relationship remain unclear. Existing reviews have combined pregnant and postpartum women, merged heterogeneous intervention types, and have not evaluated how exercise intensity, duration, frequency, or programme length modify outcomes (10). Crucially, no synthesis has identified the aerobic exercise dose associated with optimal improvements in postpartum depression, anxiety, or quality of life. These gaps limit the development of precise, feasible, and evidence-based recommendations for postpartum women.

Therefore, this study quantified the effects of aerobic exercise on postpartum depressive symptoms, anxiety, and HRQoL, and used dose–response modelling to identify the optimal weekly aerobic exercise dose for these outcomes.

## 2 Methods

### 2.1 Protocol and registration

This systematic review and meta-analysis followed the Preferred Reporting Items for Systematic Reviews and Meta Analyses (PRISMA) statement and its updated guidance for quantitative syntheses (12). The protocol was prospectively registered in the International Prospective Register of Systematic Reviews.

### 2.2 Search strategy

A comprehensive literature search was performed in PubMed, Embase, the Cochrane Central Register of Controlled Trials, Web of Science, and Google Scholar from database inception (earliest available indexing date) through 31 October 2025; the final search update was completed on 31 October 2025. No language restrictions were applied at the search stage. The strategy combined controlled vocabulary and free text terms related to the postpartum period, depression, anxiety, quality of life, aerobic exercise, jogging, and physical activity. Search strings were adapted to each database and are provided in Supplementary file 1, together with the complete search dates. Reference lists of relevant trials and prior reviews were manually screened to identify additional eligible studies. After duplicate removal in EndNote X9, two reviewers independently screened titles and abstracts, followed by full text assessment. Disagreements were resolved by consensus, and when necessary adjudicated by a third reviewer.

### 2.3 Eligibility criteria and study selection

Studies were included if they met the following PICOS criteria. (a) Participants were women aged 18 years or older who were either pregnant or in the postpartum period, irrespective of parity, delivery mode, or baseline symptom status. (b) Interventions consisted of structured aerobic exercise programmes, defined according to WHO guidance as rhythmic and continuous activities involving large muscle groups over a sustained period. Eligible programmes could be supervised or unsupervised, delivered in laboratory or real-world settings, including home based or remotely guided formats, provided that aerobic exercise was the primary active component. (c) Comparators were inactive or minimal intervention conditions, including no treatment, wait list, education or attention control, or usual care. (d) Outcomes had to include at least one validated measure of depressive symptoms, anxiety symptoms, or health related quality of life assessed during pregnancy or postpartum at the end of intervention and or follow up. (e) Only randomized controlled trials (RCTs) (13) published in peer reviewed journals were eligible. To focus on training interventions and exclude single-bout studies, programmes were required to last at least four weeks.

Studies were excluded if the intervention evaluated only acute responses to a single exercise bout, if aerobic exercise could not be isolated due to multi component programmes dominated by non-exercise elements such as diet or sleep manipulation, or if the exercise arm combined multiple lifestyle components without a clearly separable aerobic dose. Trials were also excluded when outcome data were insufficient for effect size estimation and could not be obtained from authors, when full texts were unavailable, or when the report was a conference abstract, protocol, animal study, or other non-peer reviewed publication. If multiple reports described the same trial, the most complete dataset was used.

### 2.4 Data extraction and management

Two reviewers independently extracted data using a piloted standardized form. Extracted information included publication characteristics, study design, setting, sample size, participant demographics (including parity, where reported), postpartum or pregnancy stage, baseline symptom severity, baseline BMI (where reported), intervention and comparator details, adherence, and follow up duration. For each outcome, means and standard deviations at baseline and post intervention or follow up were extracted for both groups. When standard errors were reported, standard deviations were derived as SD = SE × √n. When standard deviations were missing, they were imputed using methods recommended in the Cochrane Handbook, based on confidence intervals, t statistics, *p* values, interquartile ranges, or ranges as appropriate. Authors were contacted at least four times over six weeks to obtain unavailable data. Exercise characteristics relevant to dose response analyses were collected, including session frequency, intensity, and duration. Intensity was extracted as reported (e.g., %HRmax, %HRR, VO_2_max, or Borg RPE) to support MET assignment. When intensity was reported using HR, VO2max, or RPE targets, it was converted to corresponding MET values; when not reported, METs were assigned based on the reported exercise mode and session details using the 2011 Compendium of Physical Activities (14). Baseline depression status was recorded when available. Studies with missing baseline data were retained in the main meta-analysis and dose–response models but categorized as “NA” only in subgroup analyses, recognizing that this may increase heterogeneity and misclassification in subgroup estimates.

### 2.5 Outcomes

The primary outcomes were depressive symptoms, anxiety symptoms, and HRQoL measured postpartum or during pregnancy at the end of intervention and at the longest follow-up. Outcomes were derived from validated scales and reflect symptom severity rather than clinical diagnosis unless explicitly assessed. When multiple scales were reported, observer-rated instruments were preferred, and for depression, the most commonly used scale across trials was selected to reduce heterogeneity.

### 2.6 Risk of bias assessment

Risk of bias was assessed independently by two reviewers using the revised Cochrane Risk of Bias tool for randomized trials (RoB 2) (15). The evaluation covered bias arising from the randomization process, deviations from intended interventions, missing outcome data, outcome measurement, and selective reporting. Any disagreements were resolved through discussion or consultation with a third reviewer. Overall risk of bias for each outcome was derived according to RoB 2 guidance.

### 2.7 Aerobic exercise dose coding

Aerobic exercise dose was quantified as metabolic equivalent task minutes per week. One metabolic equivalent of task (MET) represents the energy expenditure at rest, and MET minutes per week reflect the product of exercise intensity and total weekly duration. This metric aligns with World Health Organization physical activity guidelines, which recommend at least 600 MET minutes per week for substantial health benefits in adults. For each trial arm, weekly MET minutes were calculated as the product of session duration, weekly frequency, and intensity expressed in METs. When intensity was not directly reported, it was estimated from activity type or prescribed workload using established compendium and trial descriptions (14). Where intensity targets were provided (e.g., %HRmax, %HRR, VO_2_max, or RPE), these were used to inform MET assignment for harmonization across trials. Specifically, when intensity was explicitly reported (HR, VO2max, or RPE), we converted it to METs; when intensity was not reported, we mapped the described activity and workload to the closest MET code in the 2011 Compendium of Physical Activities. Potential error may arise from nonspecific activity descriptions and inter-individual variability in energy expenditure (14). To enhance network connectivity for dose response modelling, doses were rounded to the nearest 250 MET minutes per week and classified into ordinal categories of 250, 500, 750, 1,000, and 1,250 MET minutes per week (16).

### 2.8 Statistical analyses

Pairwise meta-analyses were performed in Stata version 17. Random effects models were used *a priori* to account for between study variability in participants and measurement tools. Effect sizes were calculated as standardized mean differences with 95 percent confidence intervals, based on change scores from baseline to post intervention. Change means were computed as post intervention minus baseline values. When change score standard deviations were not available, they were derived using SD_change = √(SD_baseline^2^ + SD_post^2^ − 2R × SD_baseline × SD_post), assuming a conservative within participant correlation of R = 0.5 (17). Negative standardized mean differences indicated improvement relative to control. Statistical heterogeneity was assessed using Cochran Q and quantified with the I^2^ statistic, with values of 25, 50, and 75% interpreted as low, moderate, and high heterogeneity (18). Publication bias was evaluated by visual inspection of funnel plots and by Egger regression, with *p* values below 0.05 indicating small study effects (19). Sensitivity analyses were conducted by excluding outliers defined as trials whose 95 percent confidence intervals did not overlap the pooled 95 percent confidence interval. Prespecified subgroup analyses explored potential moderators of effect, including age, baseline depression status, intervention stage, intervention type, intensity, session time, weekly frequency, intervention duration, and adherence level.

Dose response relationships were examined in R version 4.3.0 using the MBNMAdose package to implement a model-based network meta-analysis within a Bayesian random effects framework (20). Network connectivity, model convergence, and consistency between direct and indirect evidence were evaluated using standard diagnostics and graphical assessments. Competing linear and nonlinear dose response functions were compared using deviance information criterion, residual deviance, between study standard deviation, and model parsimony (21). Restricted cubic splines provided the best balance of fit and interpretability and were therefore selected to characterize potential nonlinear associations between aerobic exercise dose and each outcome.

## 3 Results

### 3.1 Characteristics of included studies

**Figure 1:**
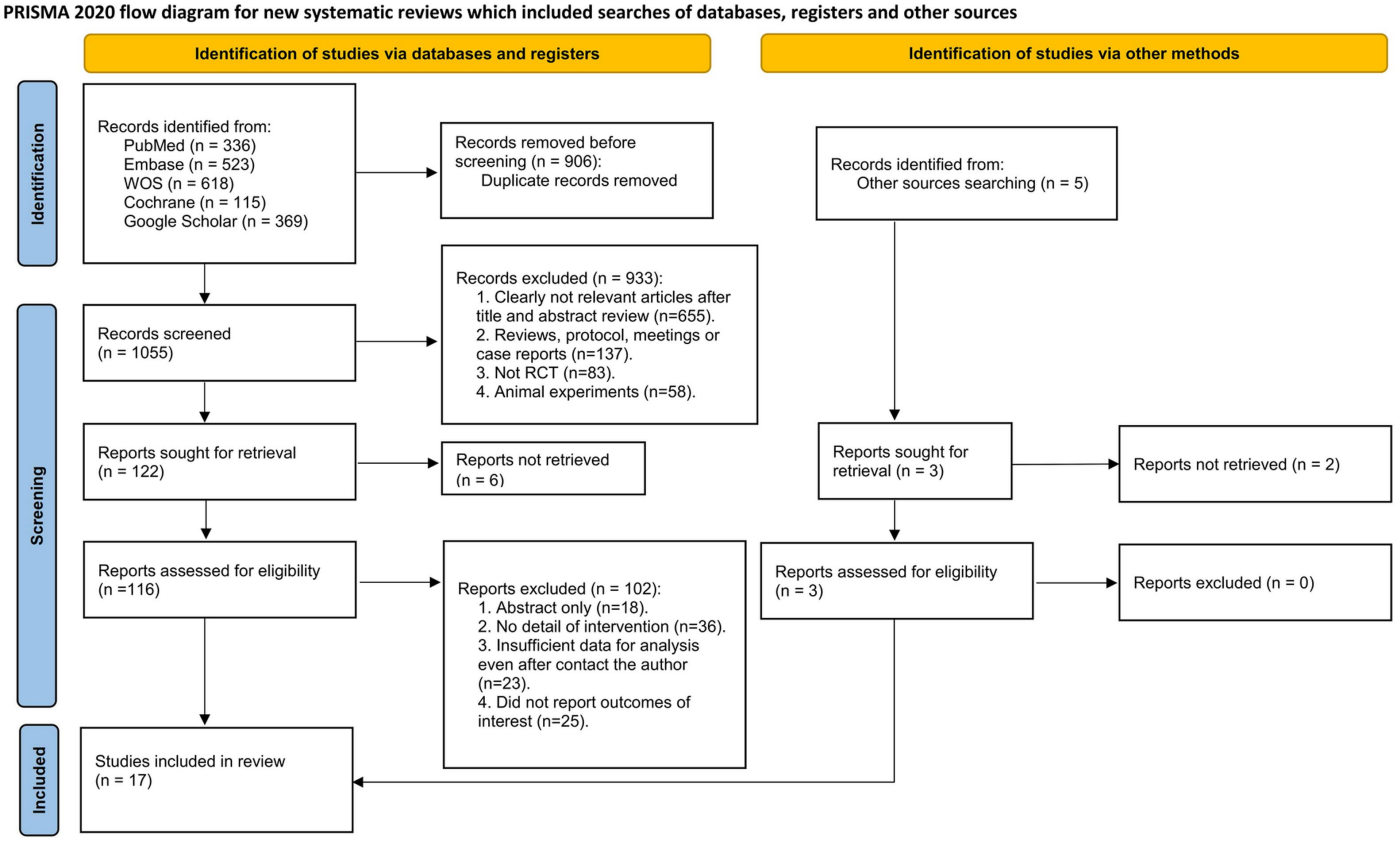
PRISMA flow diagram of study identification, screening, eligibility assessment, and inclusion.

A total of 1961 records were identified through the initial electronic search. After removing 906 duplicate records, 1,055 articles were screened based on title and abstract. Following the exclusion of 933 studies, 122 articles were further assessed for eligibility, ultimately leading to the inclusion of 17 RCTs with 2,865 postpartum women in the systematic review and network meta-analysis (Figure 1) (22–38).

The included studies were published between 2003 and 2024, with a median publication year of 2018. Sample sizes varied from 19 to 784 participants, with a median of 88 participants per study. The mean age of participants ranged from 25.0 to 34.3 years, with a median of 30.2 years. Fifteen studies reported baseline depression scores for participants, with 10 studies reporting baseline depression scores above the threshold for clinical depression. Regarding the interventions, 9 studies employed aerobic exercise, while 8 combined aerobic exercise with resistance training. Exercise intensity ranged from 4.0 METs to 5.5 METs, with a median of 4.3 METs. Session durations varied between 30 and 60 min, with a median of 35 min per session. Exercise frequency ranged from 1 to 5 times per week, with a median of 3 sessions per week. Intervention durations ranged from 4 to 26 weeks, with a median of 12 weeks. Of the included studies, 5 focused on interventions during pregnancy, and 12 were conducted postpartum. Parity was reported in 10 trials, with the proportion of primiparous women ranging from approximately 41 to 74%; baseline BMI was reported in 7 trials, with mean values ranging from 23.0 to 35.1 kg/m^2^ (Supplementary Table 4). Detailed characteristics of the included studies are presented in Supplementary file 3.

### 3.2 Meta-analysis results

#### 3.2.1 Depression

**Figure 2:**
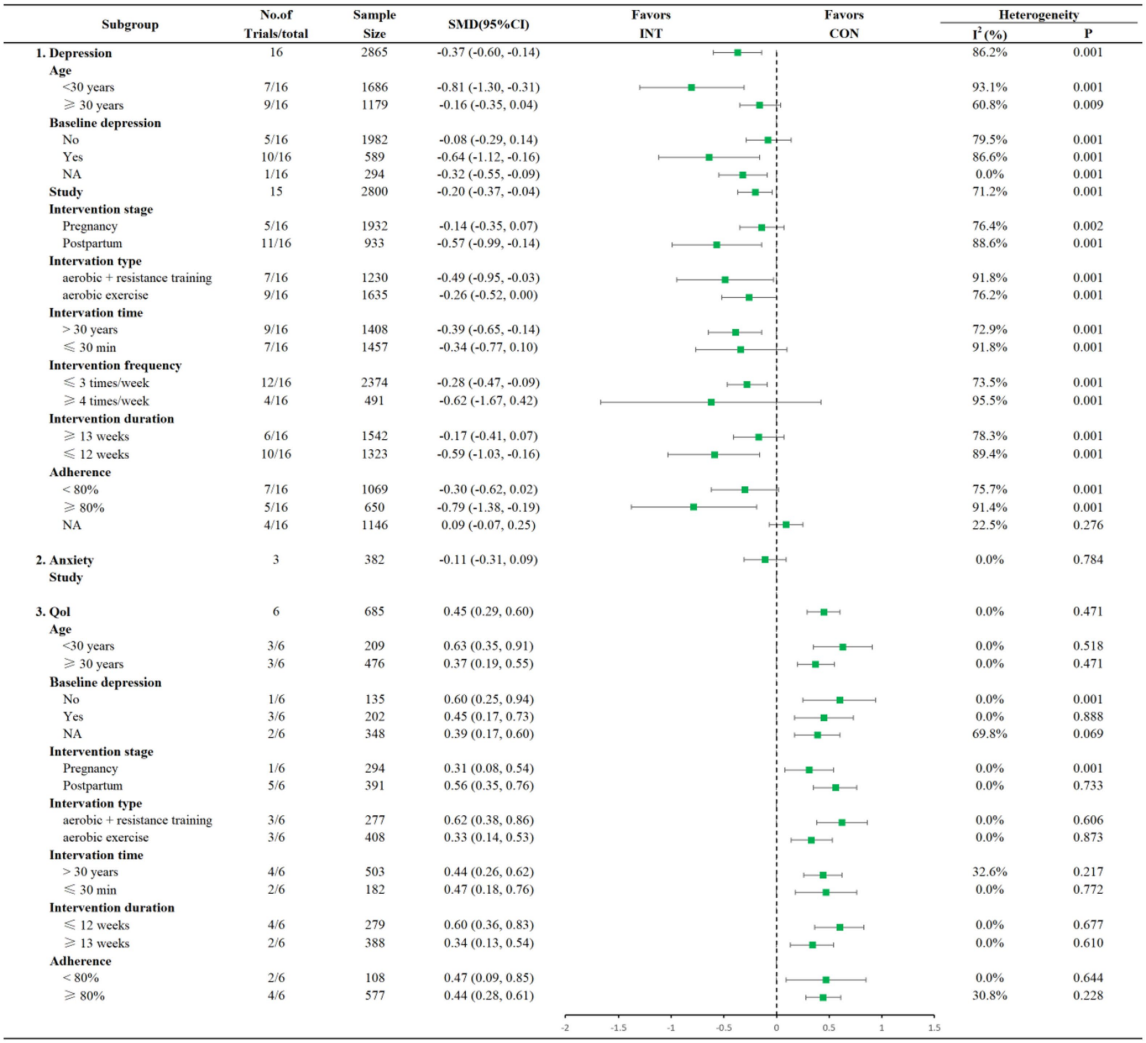
Forest plots of pairwise meta-analysis and subgroup analyses examining the effects of aerobic exercise on postpartum depressive symptoms (2.1), postpartum anxiety (2.2), and postpartum quality of life (2.3).

Sixteen studies reported the effects of exercise interventions on postpartum depression symptoms. As shown in Figure 2, aerobic exercise demonstrated a significant overall effect on improving postpartum depression (SMD = −0.37, 95% CI = −0.60 to −0.14). Subgroup analysis revealed variations in depression improvement across different factors (age, baseline depression, intervention stage, intervention time, frequency, duration, and adherence). Specifically, aerobic exercise significantly improved depression symptoms in women aged <30 years (SMD = −0.81, 95% CI = −1.30 to −0.31), but no significant effect was found in women aged ≥30 years. Similarly, significant improvements were observed in women with baseline depression (SMD = −0.64, 95% CI = −1.12 to −0.16) and those with missing baseline data (SMD = −0.32, 95% CI = −0.55 to −0.09), whereas no significant improvement was noted in women without baseline depression. Postpartum interventions showed a greater effect (SMD = −0.57, 95% CI = −0.99 to −0.14) compared to interventions during pregnancy, which were not significant. Exercise sessions longer than 30 min had a significant effect (SMD = −0.39, 95% CI = −0.65 to −0.14), while sessions of ≤30 min did not. A frequency of ≤3 sessions per week was associated with significant improvements (SMD = −0.28, 95% CI = −0.47 to −0.09), while more frequent interventions (≥4 sessions/week) did not show significant effects. Interventions lasting ≤12 weeks were significantly effective (SMD = −0.59, 95% CI = −1.03 to −0.16), while those lasting ≥13 weeks did not demonstrate significant effects. Adherence ≥80% was associated with significant improvements (SMD = −0.79, 95% CI = −1.38 to −0.19), whereas adherence <80% or NA showed no significant effects. Sensitivity analysis indicated robust results, confirming the stability of the findings (Supplementary Figure S5.1).

**Figure 3:**
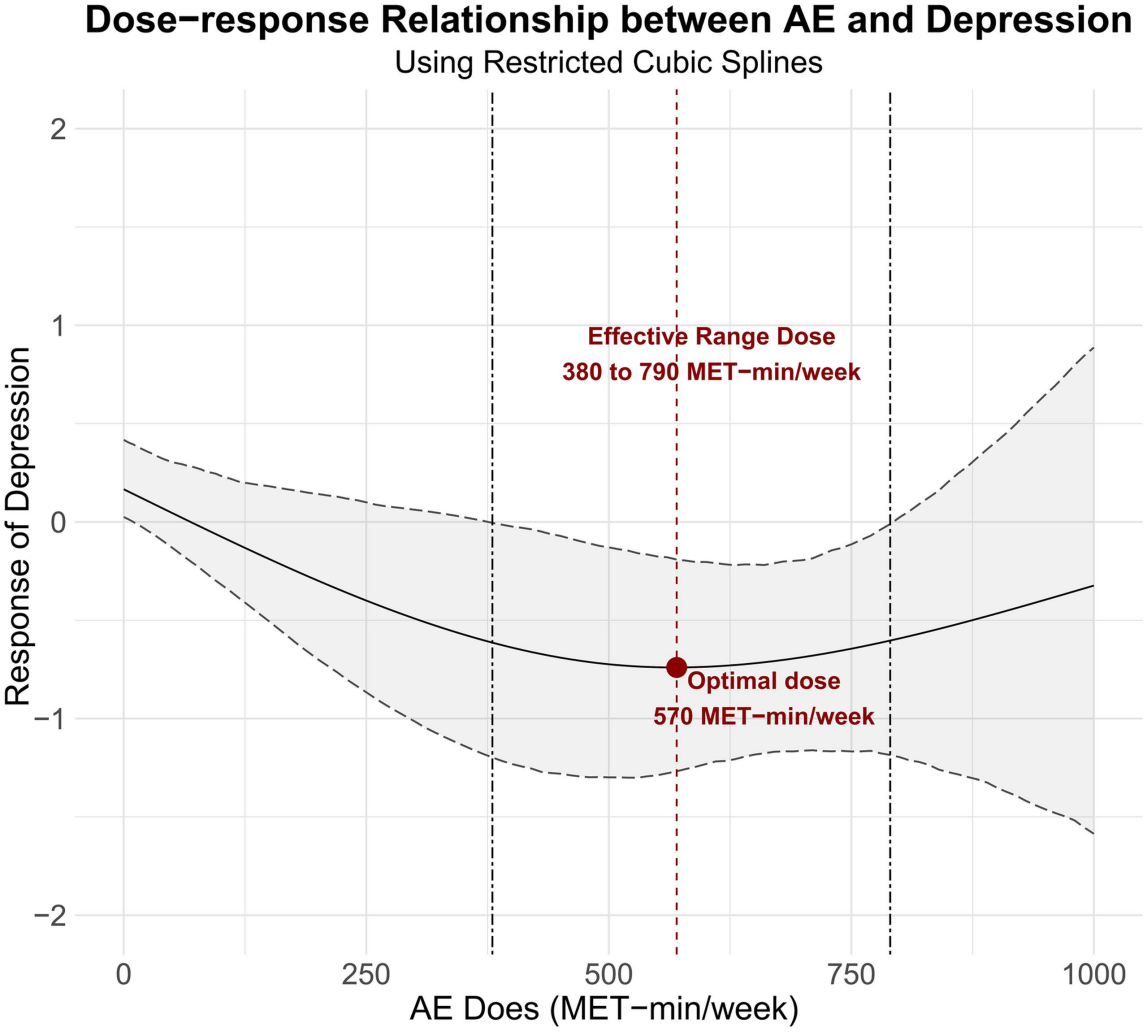
Dose–response relationship between aerobic exercise volume and improvements in postpartum depressive symptoms.

A dose–response analysis revealed a U-shaped relationship between aerobic exercise dose and postpartum depression improvement (Figure 3). Specifically, depression symptoms improved with weekly doses of 380 MET-min to 790 MET-min, with the optimum dose being around 570 MET-min/week.

#### 3.2.2 Anxiety

Three studies assessed the impact of aerobic exercise on postpartum anxiety. As shown in Figure 2, aerobic exercise did not show a significant effect on reducing anxiety (SMD = −0.11, 95% CI = −0.31 to 0.09). Due to the limited number of studies, subgroup and dose–response analyses were not performed for anxiety. Sensitivity analysis results were consistent, with no evidence of significant effects (Supplementary Figure S5.2).

#### 3.2.3 Quality of life (QoL)

Six studies reported the effects of aerobic exercise on postpartum quality of life. The meta-analysis demonstrated a significant improvement in QoL following aerobic exercise interventions (SMD = 0.45, 95% CI = 0.29 to 0.60), as shown in Figure 2. No significant differences were found across subgroups, indicating that aerobic exercise effectively improved QoL in all groups. Sensitivity analysis confirmed the stability of the findings (Supplementary Figure S5.3).

**Figure 4:**
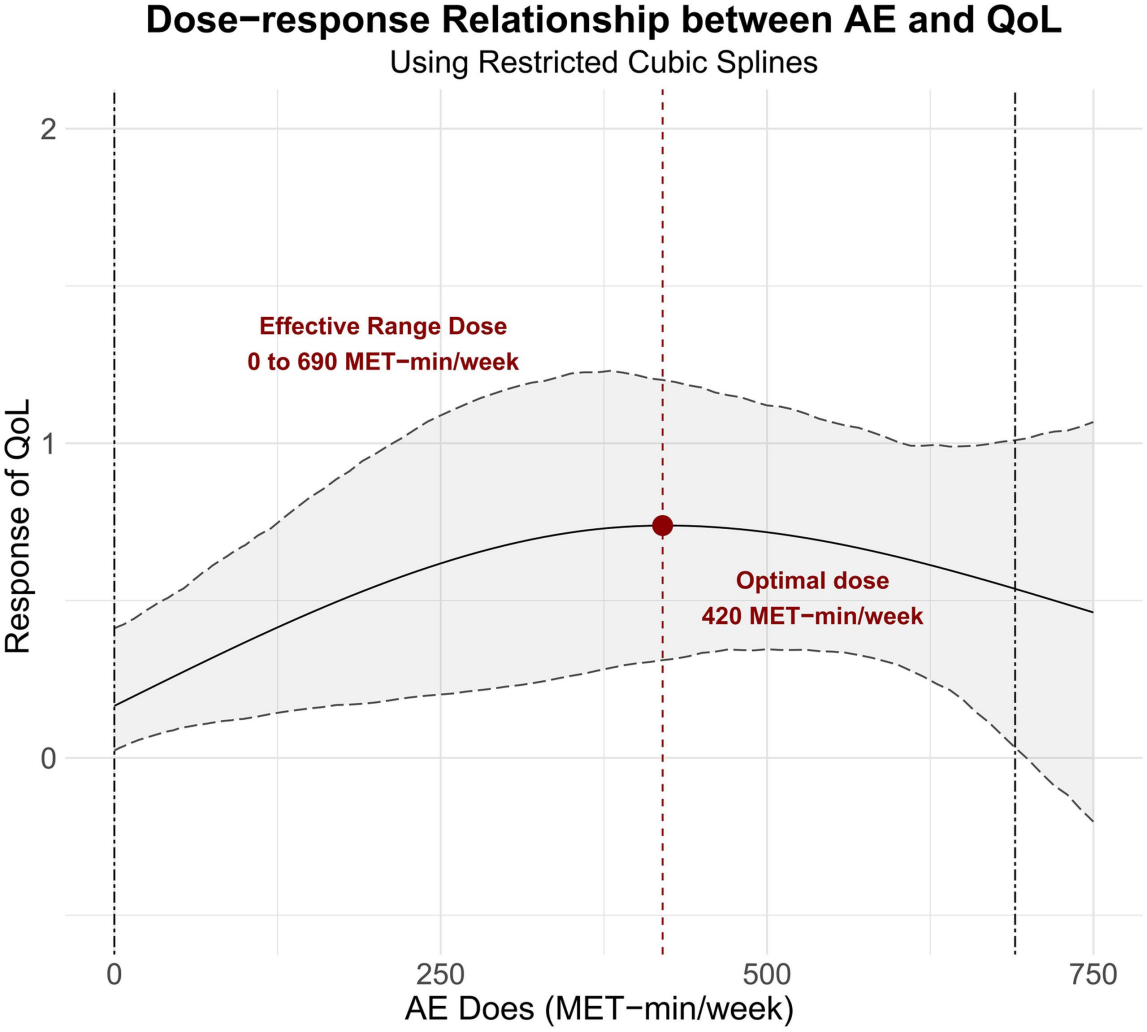
Dose–response relationship between aerobic exercise volume and improvements in postpartum quality of life.

A dose–response relationship for QoL was observed (Figure 4). The effect of aerobic exercise on QoL followed an inverted U-shaped dose–response curve. Specifically, aerobic exercise was most effective at improving QoL between 0 MET-min/week and 690 MET-min/week, with the optimal dose being 420 MET-min/week.

### 3.3 Risk of bias and publication bias

Among the 17 included RCTs, 10 were assessed as having low risk of bias overall, while 7 studies were deemed to have some concerns. In terms of randomization, 15 studies showed low risk of bias, while 2 had some concerns. Bias related to deviations from intended interventions was low in 12 studies and some concerns in 5. Missing outcome data posed low risk of bias in 12 studies and some concerns in 5. All studies had low risk of bias in outcome measurement and selective reporting (Supplementary file 4). Overall low-risk judgments were mainly driven by adequate randomization procedures, outcome measurement using validated instruments, and low risk of selective reporting. Studies rated as having some concerns most commonly reflected deviations from intended interventions and incomplete outcome data.

Funnel plots were used to assess potential publication bias (Supplementary Figure S6). The plots showed varying degrees of asymmetry, suggesting possible publication bias. Specifically, the funnel plots for depression (Supplementary Figures S6.1–6.3) exhibited asymmetry. Egger’s test for depression yielded *p* = 0.006, indicating potential publication bias. Other outcomes did not show significant publication bias, as Egger’s test results for anxiety and QoL were all >0.05.

## 4 Discussion

### 4.1 Main findings

This meta-analysis synthesized evidence from 17 RCTs including 2,865 postpartum women to evaluate the effects of aerobic exercise on depressive symptoms, anxiety, and health-related quality of life. Aerobic exercise significantly reduced postpartum depressive symptoms and improved HRQoL. Dose–response modelling indicated maximal benefit at approximately 570 MET-min per week for depressive symptoms and 420 MET-min per week for HRQoL, supporting a practical weekly target of about 400 to 600 MET-min in postpartum care. Evidence for anxiety remained limited and did not show a significant effect across three trials. Overall, these findings support aerobic exercise as a feasible and scalable strategy for postpartum mental health, with dose and programme delivery likely shaping the magnitude of benefit.

### 4.2 Mechanisms and biological plausibility

Postpartum depressive symptoms are a central determinant of maternal recovery because they affect mood, motivation, caregiving capacity, and the quality of mother infant interactions, with downstream consequences for child development and family functioning. Within this context, the present meta-analysis shows that aerobic exercise is associated with a meaningful reduction in postpartum depressive symptoms, and that effects vary by participant profile and intervention delivery. Across 16 trials, the pooled effect size indicated a moderate improvement in depressive symptoms compared with inactive or minimal control conditions, and larger gains were observed in younger women, in those who already met or approached clinical thresholds at baseline, and when interventions were delivered after childbirth rather than during pregnancy. In contrast, women without baseline depressive symptoms may show smaller changes because symptom scores are often low at baseline, leaving limited room for improvement and reducing apparent responsiveness. Studies with missing baseline depression information were retained in the primary models and classified as “missing” only for subgroup comparisons; consequently, this subgroup likely combines heterogeneous baseline severity and should be interpreted cautiously. These findings are broadly in line with previous syntheses of physical activity for perinatal depression, which have also reported small to moderate effect sizes and greater benefits among women with elevated baseline symptoms, but the present work adds precision by isolating aerobic exercise and by identifying specific prescription characteristics associated with larger effects (10, 39). The pattern of subgroup results is biologically and clinically plausible. Aerobic exercise can improve depressive symptoms through multiple pathways, including enhancement of monoamine and endorphin signaling, upregulation of brain derived neurotrophic factor, modulation of hypothalamic pituitary adrenal axis activity, and reduction of low-grade inflammation, alongside behavioral mechanisms such as better sleep, increased self-efficacy, and structured respite from caregiving demands (40). In postpartum women, these behavioral pathways may be particularly relevant given common sleep disruption and caregiving-related stress, and supervised or group-based programmes may also support stress management and social interaction (41). Biologically, the postpartum period involves marked hormonal fluctuations and recovery-related inflammatory changes, which may heighten vulnerability to mood symptoms; exercise-related effects on stress regulation and inflammatory pathways are therefore aligned with postpartum physiology (42). Women with higher baseline symptom burden and younger age may have more physiological and psychosocial capacity for change, and therefore exhibit larger standardized improvements (43). Interventions delivered in the postpartum period may also be better timed to address the peak incidence of depressive episodes and the specific stressors of early motherhood.

### 4.3 Clinical implications

Programme characteristics appear to shape these mechanisms. Sessions lasting longer than 30 min and interventions of up to 12 weeks are likely to provide a sufficient cumulative stimulus for neurobiological and psychological adaptation, whereas more frequent or prolonged programmes may increase time burden, fatigue, and dropout in women already facing substantial role strain. The strong gradient by adherence further supports the view that regular exposure to the exercise stimulus is crucial for achieving and maintaining antidepressant effects (9). Building on this, the dose response analysis offers quantitative insight into how much aerobic exercise is needed. The observed U-shaped association, with clear benefits between about 380 and 790 MET minutes per week and a peak effect near 570 MET minutes per week, suggests that both very low and very high weekly volumes are less effective. At the lower end, the stimulus may be insufficient to induce sustained changes in neurotrophic, inflammatory, and stress regulatory pathways. At higher volumes, accumulated fatigue, musculoskeletal discomfort, competition with caregiving and sleep, and the perception of exercise as an additional stressor may blunt mood benefits and undermine adherence (44, 45). An intermediate weekly volume may therefore provide the optimal balance between activating beneficial biological pathways and maintaining feasibility in the postpartum context, reinforcing moderate, structured prescriptions as the most practical approach for symptom reduction.

Anxiety symptoms represent another important component of postpartum mental health, often co-occurring with depression and contributing independently to impaired functioning, heightened physiological arousal, and increased risk of long-term anxiety disorders. Unlike the consistent antidepressant effects observed in this review, the evidence for anxiolytic benefits of aerobic exercise in postpartum women remains inconclusive. Across the three available trials, the pooled effect did not reach statistical significance, and sensitivity analyses yielded similar results, indicating that current data are insufficient to confirm a reliable anxiolytic effect. These findings partly diverge from meta-analyses in non-postpartum populations, where aerobic exercise has shown small to moderate reductions in generalized anxiety symptoms (46). However, they align with several perinatal-focused reviews that have reported either weak or inconsistent anxiolytic effects (8). The discrepancy may reflect meaningful differences in anxiety pathophysiology during the postpartum period. Postpartum anxiety often involves heightened vigilance regarding infant safety, fluctuating hormonal states, disrupted sleep, and caregiving demands that may not respond as readily to exercise-induced physiological adaptations (47). Whereas exercise may improve mood through neurotrophic, monoaminergic, and inflammatory pathways, anxiety may be more strongly driven by cognitive and situational stressors, such as intrusive worries, maternal role adjustment, and perceived lack of control, which may require more targeted psychological strategies. Several factors may also explain the absence of detectable anxiolytic effects in the current evidence base. First, postpartum anxiety symptoms are heterogeneous and may not be captured adequately by the scales used in existing trials (48). Second, the included interventions may not have reached the intensity, duration, or contextual relevance needed to influence anxiety-specific mechanisms, such as autonomic regulation or cognitive threat appraisal. Third, the small sample size and limited number of trials reduce statistical power. Longer follow-up may also be required to detect anxiety-related changes.

Quality of life is a core dimension of postpartum recovery because it reflects women’s physical functioning, emotional well-being, social participation, and overall capacity to adapt to the demands of early motherhood. In this review, aerobic exercise was associated with a meaningful improvement in postpartum quality of life, with a moderate pooled effect and no significant variation across subgroups. This consistency suggests that the benefits of aerobic exercise extend broadly across different ages, baseline mental health profiles, and intervention formats, indicating a level of generalizability not always observed for depressive or anxiety outcomes. These findings align with previous research in perinatal and non-perinatal populations, which has consistently linked structured aerobic activity to higher HRQoL scores, particularly in domains reflecting vitality and physical functioning (49). The present results contribute additional clarity by demonstrating that aerobic exercise alone, rather than multimodal activity programmes, is sufficient to yield measurable improvements in postpartum quality of life. Aerobic exercise enhances cardiorespiratory fitness, muscular endurance, and energy availability, which may directly improve women’s ability to perform daily tasks and engage in caregiving (50). Exercise is also known to increase sleep quality, reduce perceived stress, and promote a sense of mastery and self-efficacy, all of which contribute to broader quality-of-life dimensions (51). Compared with depressive or anxiety symptoms, quality of life encompasses multiple physical and psychosocial domains that may be responsive to even modest improvements in fitness, daily functioning, and emotional regulation. This may explain why significant benefits were observed regardless of age, baseline symptom status, or intervention characteristics: women with varying levels of psychological burden may nonetheless experience tangible functional gains that translate into improved life quality. The dose–response findings further refine this interpretation. The inverted U-shaped association, with progressive gains up to approximately 420 MET minutes per week and diminishing returns beyond roughly 690 MET minutes per week, suggests that moderate aerobic activity may optimize functional and psychological improvements without imposing excessive physical or logistical burden. At moderate doses, exercise likely enhances physical capacity, stimulates endorphin release, improves cardiovascular and metabolic function, and supports social or behavioral activation, all of which contribute to enhanced quality of life. At higher doses, however, that is, higher weekly exercise volumes in MET-min/week driven by longer sessions and or greater weekly frequency (and, in some programmes, higher prescribed intensity), cumulative fatigue, time constraints, musculoskeletal strain, and interference with sleep or caregiving may offset these benefits (52). The identification of an optimal dose range reinforces the principle that postpartum exercise prescriptions should prioritize feasibility and sustainability, given common time constraints, childcare responsibilities, ongoing physical recovery, and sleep disruption in the early postpartum period.

### 4.4 Limitations and future directions

This study has several notable strengths. First, it is the most comprehensive synthesis to date focusing exclusively on aerobic exercise in the postpartum period, allowing for a clearer attribution of effects than prior reviews that pooled heterogeneous exercise modalities or combined pregnant and postpartum populations. Second, the use of model-based dose response analysis provided quantitative estimates of optimal aerobic exercise volumes for improving depressive symptoms and quality of life, offering practical guidance for clinical and public health recommendations. Several limitations should also be acknowledged. First, although the overall sample was large, the evidence base for postpartum anxiety remained limited to three small trials, restricting the precision of pooled estimates and precluding subgroup or dose response analyses for this outcome. Second, heterogeneity in intervention reporting, especially intensity prescribed using different metrics, required estimation of MET values in some studies, which may introduce uncertainty into dose response modelling. In studies without explicit intensity reporting, METs were assigned based on the reported activity mode and session details using the 2011 Compendium of Physical Activities, which may introduce misclassification error when exercise descriptions are nonspecific. Relatedly, most interventions clustered within a moderate-intensity range, limiting intensity-stratified analyses. Therefore, effects attributed to programme characteristics such as session duration may partly reflect differences in effective intensity or accumulated load. Third, key maternal characteristics, including parity and baseline BMI, were inconsistently reported across trials, limiting assessment of their potential influence on intervention effects. Fourth, despite rigorous search procedures, potential publication bias was detected for depression outcomes, suggesting that the magnitude of benefit may be overestimated and highlighting the need for future trials with pre-registered protocols and transparent reporting. Together, these limitations indicate that although the present findings provide important insights, further high-quality randomized trials with standardized intervention reporting including clear intensity specification are warranted to strengthen the evidence base for postpartum exercise prescriptions.

## 5 Conclusion

Aerobic exercise effectively improves postpartum mental health, moderately reducing depressive symptoms and enhancing quality of life. Dose–response modelling showed maximal benefits at approximately 570 MET-min/week for depression and 420 MET-min/week for quality of life. In practice, 400 to 600 MET-min/week is roughly 100 to 150 min per week of moderate-intensity activity (about 4 METs), such as brisk walking, completed as 30 to 45 min on 2 to 4 days per week or in shorter bouts to fit childcare demands and recovery. No clear anxiolytic effect was observed due to limited data. Prescribing exercise within this range offers a feasible, evidence-based strategy for postpartum care, while further trials are needed to clarify effects on anxiety and refine dose recommendations.

## Data Availability

The original contributions presented in the study are included in the article/Supplementary material, further inquiries can be directed to the corresponding author.
