# Supplementary material for: Optimal dose of aerobic exercise for improving postpartum depression, anxiety, and quality of life: a meta-analysis of randomized controlled trials and dose–response analysis
Source: Front Public Health. 2026 Apr 21;14:1807903. doi: 10.3389/fpubh.2026.1807903 (PMC13139009; doi:10.3389/fpubh.2026.1807903)
Supplement: Supplementary file 1 [file Supplementary_file_1.DOCX]

**Supplementary**

Table of Contents:

[Supplementary 1: PRISMA Checklist 1](#_Toc534204563)

[Supplementary 2: Search Strategy 4](#_Toc1894445881)

[Supplementary 3: Characteristics of studies and subjects included in the review 6](#_Toc1347370016)

[Supplementary 4: Risk of Bias 1](#_Toc1262935083)0

[Supplementary 5: Sensitivity analysis 1](#_Toc431254745)1

[Supplementary 6: Publication bias 1](#_Toc431254745)3

**Supplementary 1: PRISMA Checklist**

| Item # | Checklist Item | Reported on Page # |
| --- | --- | --- |
|  |  | 1 |
| 1 | Identify the report as a systematic review incorporating a network meta-analysis (or related form of meta-analysis). | 1 |
| 2 | Provide a structured summary including, as applicable:  **Background:** main objectives  **Methods:** data sources; study eligibility criteria, participants, and interventions; study appraisal; and *synthesis methods, such as network meta-analysis.*  **Results:** number of studies and participants identified; summary estimates with corresponding confidence/credible intervals; treatment rankings may also be discussed. Authors may choose to summarize pairwise comparisons against a chosen treatment included in their analyses for brevity.  **Discussion/Conclusions:** limitations; conclusions and implications of findings.  **Other:** primary source of funding; systematic review registration number with registry name. | 1-2 |
| 3 | Describe the rationale for the review in the context of what is already known*, including mention of why a meta-analysis of randomized controlled trials and dose-response analysis has been conducted.* | 3-5 |
| 4 | Provide an explicit statement of questions being addressed, with reference to participants, interventions, comparisons, outcomes, and study design (PICOS). | 5-6 |
| 5 | Indicate whether a review protocol exists and if and where it can be accessed (e.g., Web address); and, if available, provide registration information, including registration number. | 5 |
| 6 | Specify study characteristics (e.g., PICOS, length of follow-up) and report characteristics (e.g., years considered, language, publication status) used as criteria for eligibility, giving rationale. *Clearly describe eligible treatments included in the treatment network, and note whether any have been clustered or merged into the same node (with justification).* | 28-29 |
| 7 | Describe all information sources (e.g., databases with dates of coverage, contact with study authors to identify additional studies) in the search and date last searched. | 5 |
| 8 | Present full electronic search strategy for at least one database, including any limits used, such that it could be repeated. | Supplementary 2 |
| 9 | State the process for selecting studies (i.e., screening, eligibility, included in systematic review, and, if applicable, included in the meta-analysis). | 5-6 |
| 10 | Describe method of data extraction from reports (e.g., piloted forms, independently, in duplicate) and any processes for obtaining and confirming data from investigators. | 6-7 |
| 11 | List and define all variables for which data were sought (e.g., PICOS, funding sources) and any assumptions and simplifications made. | 7-8 |
| S1 | Describe methods used to explore the geometry of the treatment network under study and potential biases related to it. This should include how the evidence base has been graphically summarized for presentation, and what characteristics were compiled and used to describe the evidence base to readers. | 8 |
| 12 | Describe methods used for assessing risk of bias of individual studies (including specification of whether this was done at the study or outcome level), and how this information is to be used in any data synthesis. | 8, Supplementary 4 |
| 13 | State the principal summary measures (e.g., risk ratio, difference in means). Also describe the use of additional summary measures assessed, such as treatment rankings and surface under the cumulative ranking curve (SUCRA) values, as well as modified approaches used to present summary findings from meta-analyses. | 8-10 |
| 14 | Describe the methods of handling data and combining results of studies for each network meta-analysis. This should include, but not be limited to:   - Handling of multi-arm trials; - Selection of variance structure; - Selection of prior distributions in Bayesian analyses; and - Assessment of model fit. | 8-10 |
| S2 | Describe the statistical methods used to evaluate the agreement of direct and indirect evidence in the treatment network(s) studied. Describe efforts taken to address its presence when found. | Supplementary 7 |
| 15 | Specify any assessment of risk of bias that may affect the cumulative evidence (e.g., publication bias, selective reporting within studies). | 9,  Supplementary 6 |
| 16 | Describe methods of additional analyses if done, indicating which were pre-specified. This may include, but not be limited to, the following:   - Sensitivity or subgroup analyses; - Meta-regression analyses; - Alternative formulations of the treatment network; and - Use of alternative prior distributions for Bayesian analyses (if applicable). | 8-10 |
| 17 | Give numbers of studies screened, assessed for eligibility, and included in the review, with reasons for exclusions at each stage, ideally with a flow diagram. | Figure 1 |
| S3 | Provide a network graph of the included studies to enable visualization of the geometry of the treatment network. | Figure 5 |
| S4 | Provide a brief overview of characteristics of the treatment network. This may include commentary on the abundance of trials and randomized patients for the different interventions and pairwise comparisons in the network, gaps of evidence in the treatment network, and potential biases reflected by the network structure. | Supplementary 9 |
| 18 | For each study, present characteristics for which data were extracted (e.g., study size, PICOS, follow-up period) and provide the citations. | Supplementary 3 |
| 19 | Present data on risk of bias of each study and, if available, any outcome level assessment. | 11,  Supplementary 4, and Supplementary 6 |
| 20 | For all outcomes considered (benefits or harms), present, for each study: 1) simple summary data for each intervention group, and 2) effect estimates and confidence intervals. *Modified approaches may be needed to deal with information from larger networks.* | Supplementary 3 |
| 21 | Present results of each meta-analysis done, including confidence/credible intervals. In larger networks, authors may focus on comparisons versus a particular comparator (e.g. placebo or standard care), with full findings presented in an appendix. League tables and forest plots may be considered to summarize pairwise comparisons. If additional summary measures were explored (such as treatment rankings), these should also be presented. | Figure 2, Figure 3, and Figure 4 |
| S5 | Describe results from investigations of inconsistency. This may include such information as measures of model fit to compare consistency and inconsistency models, *P* values from statistical tests, or summary of inconsistency estimates from different parts of the treatment network. | Supplementary 8, and Supplementary 9 |
| 22 | Present results of any assessment of risk of bias across studies for the evidence base being studied. | 11,  Supplementary 4, and Supplementary 6 |
| 23 | Give results of additional analyses, if done (e.g., sensitivity or subgroup analyses, meta-regression analyses*, alternative network geometries studied, alternative choice of prior distributions for Bayesian analyses,* and so forth). | Figure 4,  and Figure 5 |
| 24 | Summarize the main findings, including the strength of evidence for each main outcome; consider their relevance to key groups (e.g., healthcare providers, users, and policy-makers). | 14-19 |
| 25 | Discuss limitations at study and outcome level (e.g., risk of bias), and at review level (e.g., incomplete retrieval of identified research, reporting bias). *Comment on the validity of the assumptions, such as transitivity and consistency. Comment on any concerns regarding network geometry (e.g., avoidance of certain comparisons).* | 19 |
| 26 | Provide a general interpretation of the results in the context of other evidence, and implications for future research. | 19 |
| 27 | Describe sources of funding for the systematic review and other support (e.g., supply of data); role of funders for the systematic review. This should also include information regarding whether funding has been received from manufacturers of treatments in the network and/or whether some of the authors are content experts with professional conflicts of interest that could affect use of treatments in the network. | 19 |

PICOS = population, intervention, comparators, outcomes, study design.

**Supplementary 2: Search Strategy**

## Database: PubMed <inception to October 31 2025>

***Search Strategy:***

| Search number | Query |
| --- | --- |
| 20 | (((((((((Postpartum Period[MeSH Terms]) OR (Depression, Postpartum[MeSH Terms])) OR (postpartum[Title/Abstract])) OR (puerperal[Title/Abstract])) OR (postpartal[Title/Abstract])) OR (postnatal[Title/Abstract])) OR (puerperal[Title/Abstract])) AND ((((((((((((((((Exercise[MeSH Terms]) OR (Exercise Therapy[MeSH Terms])) OR (exercise[Title/Abstract])) OR (train*[Title/Abstract])) OR ("physical activit*"[Title/Abstract])) OR ("aquatic exercis*"[Title/Abstract])) OR ("water-based exercis*"[Title/Abstract])) OR (dance[Title/Abstract])) OR ("aerobic exercise*"[Title/Abstract])) OR ("endurance training"[Title/Abstract]))) AND (((((((((((((((((((((((((depression[MeSH Terms]) OR (("depression"[Title/Abstract] OR "depressive"[Title/Abstract] OR "depressed"[Title/Abstract] OR "melancholia"[Title/Abstract] OR "dysphoria"[Title/Abstract] OR "despair"[Title/Abstract] OR "despondency"[Title/Abstract] OR "emotional depression"[Title/Abstract] OR "depressive symptom"[Title/Abstract]))) OR (Anxiety[MeSH Terms])) OR (anxiety[Title/Abstract])) OR (anxious[Title/Abstract])) OR (worry[Title/Abstract])) OR (inquietude[Title/Abstract])) OR (apprehension[Title/Abstract])) OR (restlessness[Title/Abstract])) OR (perturbation[Title/Abstract])) OR (tension[Title/Abstract])) OR (uneasiness[Title/Abstract])) OR (fear[Title/Abstract])) OR (Quality of Life[MeSH Terms])) OR (QoL[Title/Abstract])) OR ("Quality of Life"[Title/Abstract])) OR (Well-being[Title/Abstract])) OR ("Life satisfaction"[Title/Abstract])) OR ("Life quality"[Title/Abstract])) OR ("Social functioning"[Title/Abstract])) OR (emotional[Title/Abstract])) OR (mental[Title/Abstract])) OR ("Short Form 36"[Title/Abstract])) OR (SF-36[Title/Abstract])) OR (SF-12[Title/Abstract]))) AND ((randomized controlled trial[pt] OR controlled clinical trial[pt] OR randomized[tiab] OR placebo[tiab] OR clinical trials as topic[mesh:noexp] OR randomly[tiab] OR trial[ti]) NOT (animals[mh] NOT (humans[mh] AND animals[mh]))) |
| 19 | (randomized controlled trial[pt] OR controlled clinical trial[pt] OR randomized[tiab] OR placebo[tiab] OR clinical trials as topic[mesh:noexp] OR randomly[tiab] OR trial[ti]) NOT (animals[mh] NOT (humans[mh] AND animals[mh])) |
| 18 | ((((((((((((((((((((((((depression[MeSH Terms]) OR (("depression"[Title/Abstract] OR "depressive"[Title/Abstract] OR "depressed"[Title/Abstract] OR "melancholia"[Title/Abstract] OR "dysphoria"[Title/Abstract] OR "despair"[Title/Abstract] OR "despondency"[Title/Abstract] OR "emotional depression"[Title/Abstract] OR "depressive symptom"[Title/Abstract]))) OR (Anxiety[MeSH Terms])) OR (anxiety[Title/Abstract])) OR (anxious[Title/Abstract])) OR (worry[Title/Abstract])) OR (inquietude[Title/Abstract])) OR (apprehension[Title/Abstract])) OR (restlessness[Title/Abstract])) OR (perturbation[Title/Abstract])) OR (tension[Title/Abstract])) OR (uneasiness[Title/Abstract])) OR (fear[Title/Abstract])) OR (Quality of Life[MeSH Terms])) OR (QoL[Title/Abstract])) OR ("Quality of Life"[Title/Abstract])) OR (Well-being[Title/Abstract])) OR ("Life satisfaction"[Title/Abstract])) OR ("Life quality"[Title/Abstract])) OR ("Social functioning"[Title/Abstract])) OR (emotional[Title/Abstract])) OR (mental[Title/Abstract])) OR ("Short Form 36"[Title/Abstract])) OR (SF-36[Title/Abstract])) OR (SF-12[Title/Abstract]) |
| 17 | Quality of Life[MeSH Terms] |
| 16 | Anxiety[MeSH Terms] |
| 15 | depression[MeSH Terms] |
| 14 | (Exercise[MeSH Terms]) OR (Exercise Therapy[MeSH Terms])) OR (exercise[Title/Abstract])) OR (train*[Title/Abstract])) OR ("physical activit*"[Title/Abstract])) OR ("aquatic exercis*"[Title/Abstract])) OR ("water-based exercis*"[Title/Abstract])) OR (dance[Title/Abstract])) OR ("aerobic exercise*"[Title/Abstract])) OR ("endurance training"[Title/Abstract]) |
| 13 | (exercise[Title/Abstract])) OR (train*[Title/Abstract])) OR ("physical activit*"[Title/Abstract])) OR ("aquatic exercis*"[Title/Abstract])) OR ("water-based exercis*"[Title/Abstract])) OR (dance[Title/Abstract])) OR ("aerobic exercise*"[Title/Abstract])) OR ("endurance training"[Title/Abstract]) |
| 6 | Exercise Therapy[MeSH Terms] |
| 5 | Exercise[MeSH Terms] |
| 4 | ((((((Postpartum Period[MeSH Terms]) OR (Depression, Postpartum[MeSH Terms])) OR (postpartum[Title/Abstract])) OR (puerperal[Title/Abstract])) OR (postpartal[Title/Abstract])) OR (postnatal[Title/Abstract])) OR (puerperal[Title/Abstract]) |
| 3 | (postpartum[Title/Abstract])) OR (puerperal[Title/Abstract])) OR (postpartal[Title/Abstract])) OR (postnatal[Title/Abstract])) OR (puerperal[Title/Abstract]) |
| 2 | Depression, Postpartum[MeSH Terms] |
| 1 | Postpartum Period[MeSH Terms] |

# Supplementary 4: Characteristics of studies and subjects included in the review

| **Study** | **Subjects**  **(intervention/ control)** | **Mean age**  **(intervention/ control)** | **Parity (primiparous / multiparous)** | **Baseline BMI** | **Baseline depression cutoff** | **Baseline depression score** | **Intervention group** | **Control group** | **Intervention stage** | **Intensity** | **METs** | **Time**  **(minutes)** | **Frequency** | **Duration** | **Adherence** | **Outcomes** |
| --- | --- | --- | --- | --- | --- | --- | --- | --- | --- | --- | --- | --- | --- | --- | --- | --- |
| Coll et al. (2019) | 579 (192/387) | 27.2 ± 5.5 vs. 27.3 ± 5.5 | 125/67 vs. 259/128 | NA | ≥10 | 5.6 ± 3.8 vs. 6.5 ± 4.4 | Moderate-intensity aerobic + resistance training | Maintain daily activities without structured exercise | Pregnancy | RPE 12-14 | 4 | 60 min | 3 times/week | 16 weeks | The average number of completed repetitions was 27.1 ± 17.2 (56.3%) | Depression |
| Teychenne et al. (2021) | 62 (32/30) | 33.6 ± 3.7 vs. 33.0 ± 3.7 | 20/12 vs. 19/11 | NA | ≥10 | 12.1 ± 3.8 vs. 12.6 ± 3.9 | Home treadmill or spinning bike + Web application | Maintain daily activities without structured exercise | Postpartum | ≥4 METs (3500 counts/minute) | 4 | 60 min | 3 times/week | 12 weeks | 97% (31/32) | Depression, Anxiety |
| Özkan SA et al. (2020) | 65 (34/31) | 28.90 ± 4.83 | NA | NA | ≥10 | 16.41 ± 1.61 vs. 15.74 ± 2.35 | Low back/pelvic stabilization, moderate-to-vigorous aerobic, strength, and balance training | Routine postpartum care (health education, pelvic floor exercises, etc.), no structured exercise | Postpartum | NA | 4.4 | 30 min | 5 times/week | 4 weeks | 85% | Depression |
| Garnæs KK et al. (2019) | 91 (46/45) | 31.2 ± 4.1 vs. 31.2 ± 4.1 | 22/24 vs. 19/26 | 33.9 ± 3.8 vs. 35.1 ± 4.6 | ＜83 | 77.2 ± 12.1 vs. 74.0 ± 13.2 | 35 min treadmill walking (~80% VO₂max) + 25 min resistance (incl. pelvic-floor) | Standard maternal care only (no discouragement of activity) | Pregnancy | RPE 12-15 | 5.4 | 60 min | 3 times/week | 24 weeks | 50% reached per-protocol adherence | Depression |
| Lewis et al. (2021) | 300 (150/150) | 31.03 ± 4.68 vs. 29.77 ± 5.32 | 33.1% (exercise), 28.3% (wellness), 34.8% (usual care) | NA | ≥10 | 7 ± 3.5 vs. 8 ± 4.7 | 6 months of telephone-guided aerobic exercise | Routine postpartum care (no structured exercise) | Postpartum | ≥55% HRmax | 5.5 | 30 min | 5 times/week | 24 weeks | NA | Depression, Anxiety |
| Armstrong K. et al. (2003) | 20 (10/10) | 21-30 | NA | NA | ≥10 | 17.40 ± 4.65 vs. 18.40 ± 4.77 | pram-push walking | Routine postpartum care, no structured exercise | Postpartum | 40–60% VO₂max | 4 | 35 min | 3 times/week | 12 weeks | 66% (23.7/36) | Depression, Anxiety, QoL |
| Armstrong K. et al. (2004) | 19（9/10） | 21-30 | NA | NA | ≥10 | 17.25 ± 4.00 vs. 17.17 ± 4.45 | pram-push walking | 1 unstructured social support group per week (09:30–11:00 open discussion) | Postpartum | 60–75% HRmax | 4 | 35 min | 3 times/week | 12 weeks | 75% (23/36) | Depression |
| Costa et al. (2009) | 88 (46/42) | 34.3 ± 3.4 vs. 32.7 ± 4.8 | 41.3%/58.7% vs. 33.3%/66.7% | NA | ≥10 | 13.6 ± 3.6 vs. 13.6 ± 3.9 | Home exercise program following ACSM guidelines: Aerobic (60–85% HRmax, 60–120 min/wk) + strength/stretching exercises | Standard postpartum routine care | Postpartum | 60–85% HRmax | 4.3 | 30 min | 4 times/week | 12 weeks | 76.1% (35/46) | Depression |
| Daley et al. (2008) | 38 (20/18) | NA | NA | NA | ≥10 | 17.7 ± 5.2 vs. 19.2 ± 4.7 | Moderate intensity walking (pushing a stroller) 30 min/day × 5 d/wk | Continue with routine postpartum care, no additional exercise prescription | Postpartum | 50-70% HRmax | 4 | 30 min | 5 times/week | 12 weeks | NA | Depression |
| Daley et al. (2015) | 94 (47/47) | 31.7 ± 5.3 vs. 29.3 ± 5.7 | NA | 28.8 ± 5.0 vs. 28.5 ± 5.8 | ≥10 | 17.3 ± 3.0 vs. 17.5 ± 3.7 | Moderate-intensity aerobic exercise (mainly brisk walking) | Regular postpartum care + a "Looking after yourself" leaflet | Postpartum | 40-60% HRmax | 4.3 | 30 min | 3 times/week | 24 weeks | 87% (41/47) | Depression, QoL |
| Daley et al. (2018) | 784 (392/392) | 27.6 ± 6.3 vs. 27.4 ± 6.3 | NA | 26.1 ± 5.3 | ≥10 | 7.6 ± 5.3 vs. 7.7 ± 5.0 | Supervised treadmill brisk walking in hospitals/community centers | Conventional smoking cessation behavioral support during pregnancy (six 20-minute individual consultations), no exercise prescription | Pregnancy | moderate intensity | 4.3 | 30 min | 2 times/week | 8 weeks | NA | Depression |
| Dritsa et al. (2008) | 88 (46/42) | 34.3 ± 3.4 vs. 32.7 ± 3.4 | 41.3% vs 33.3% | 27.08 ± 5.67 vs 26.43 ± 4.92 | ≥10 | 13.6 ± 3.6 vs. 13.6 ± 3.9 | Personalized home aerobic exercise (with strength/stretching exercises) | No structured exercise prescription, only routine postpartum care | Postpartum | 60-85% HRmax | 4.3 | 30 min | 3 times/week | 12 weeks | 76.1%（35/46） | Depression, QoL |
| Forsyth et al. (2017) | 24 (12/12) | 25.0 ± 5.1 vs. 27.0 ± 5.5 | 7/4 vs. 5/6 | 30.0 ± 6.9 vs. 32.3 ± 7.7 | ≥10 | 17.6 ± 4.0 vs. 15.9 ± 2.9 | 150 min/wk of moderate-intensity (40–65% VO₂max) exercise (optional: 60 min of group pram-walking, facility-based aerobics class, or spontaneous exercise at home) | No structured exercise prescription, only routine postpartum care | Postpartum | 40–65% VO₂max | 4.8 | 60 min | 2-3 times/week | 12 weeks | NA | Depression |
| Navas et al. (2021) | 294 (148/146) | 31.1 ± 4.1 vs. 31.5 ± 4.2 | Primiparous 67.6% vs 69.5% | 23.5 ± 3.2 vs 23.4 ± 3.1 | NA | NA | Water aerobics class (pool temperature 28–30°C) | Usual prenatal care | Pregnancy | 55–65% HRmax | 5.3 | 45 min | 3 times/week | 20 weeks | 139/148 | Depression, QoL |
| Norman et al. (2010) | 135 (62/73) | 29.3 ± 4.0 vs. 30.1 ± 5.3 | Primiparous 68% vs 63% | NA | ≥10 | 8.00 ± 6.16 vs. 6.75 ± 5.44 | Moderate-intensity aerobic exercise such as pram walking, walking, cycling or swimming; appropriate exercises for the pelvic floor and abdominal muscles; | Mailing the same educational materials | Postpartum | NA | 5 | 60 min | 1 time/week | 8 weeks | > 85% | Depression, QoL |
| Vargas‑Terrones et al. (2018) | 124 (70/54) | 33.3 ± 2.9 vs 32.3 ± 5.0 | Primiparous 70.0% vs 74.1% | 23.0 ± 3.7 vs 23.9 ± 5.0 | ≥16 | 11.0 ± 7.7 vs 10.06 ± 6.8 | Structured comprehensive exercise during pregnancy (10min warm-up – 5min walking + 5min stretching; 25min moderate intensity aerobic dance; 10min strength training; 5min coordination/balance; 5min pelvic floor exercise; 5–10min stretching and cool-down) | Receive general nutrition and exercise advice, no structured classes | Pregnancy | 55%–60% HRR | 4.3 | 60 min | 3 times/week | 26 weeks | 69.3% | Depression |

N/A None available; MET Metabolic Equivalent of Task; RPE Rating of Perceived Exertion; HRmax Maximum Heart Rate; HRR Heart Rate Reserve; VO₂max Maximal Oxygen Uptake; HDRS Hamilton Depression Rating Scale; QoL Quality of Life; ACSM American College of Sports Medicine; HIIT High Intensity Interval Training

# Supplementary 4: Risk of Bias

## Table 4.1 The risk of bias assessment for the individual included studies

| **Study** | Randomization process | Deviations from intended interventions | Missing outcome data | Measurement of the outcome | Selection of the reported result | Overall Bias |
| --- | --- | --- | --- | --- | --- | --- |
| Coll et al. (2019) | Some concerns | Some concerns | Some concerns | Low | Low | Some concerns |
| Teychenne et al. (2021) | Low | Some concerns | Low | Low | Low | Some concerns |
| Özkan SA et al. (2020) | Low | Low | Low | Low | Low | Low |
| Garnæs KK et al. (2019) | Low | Low | Low | Low | Low | Low |
| Lewis et al. (2021) | Low | Low | Low | Low | Low | Low |
| Armstrong K. et al. (2003) | Low | Some concerns | Some concerns | Low | Low | Some concerns |
| Armstrong K. et al. (2004) | Low | Low | Low | Low | Low | Low |
| Costa et al. (2009) | Low | Some concerns | Low | Low | Low | Some concerns |
| Daley et al. (2008) | Low | Low | Low | Low | Low | Low |
| Daley et al. (2015) | Low | Some concerns | Some concerns | Low | Low | Some concerns |
| Daley et al. (2018) | Some concerns | Low | Some concerns | Low | Low | Some concerns |
| Dritsa et al. (2008) | Low | Low | Low | Low | Low | Low |
| Anany et al. (2024) | Low | Low | Low | Low | Low | Low |
| Forsyth et al. (2017) | Low | Low | Some concerns | Low | Low | Some concerns |
| Navas et al. (2021) | Low | Low | Low | Low | Low | Low |
| Norman et al. (2010) | Low | Low | Low | Low | Low | Low |
| Vargas‑Terrones et al. (2018) | Low | Low | Low | Low | Low | Low |

# Supplementary 5: Sensitivity analysis


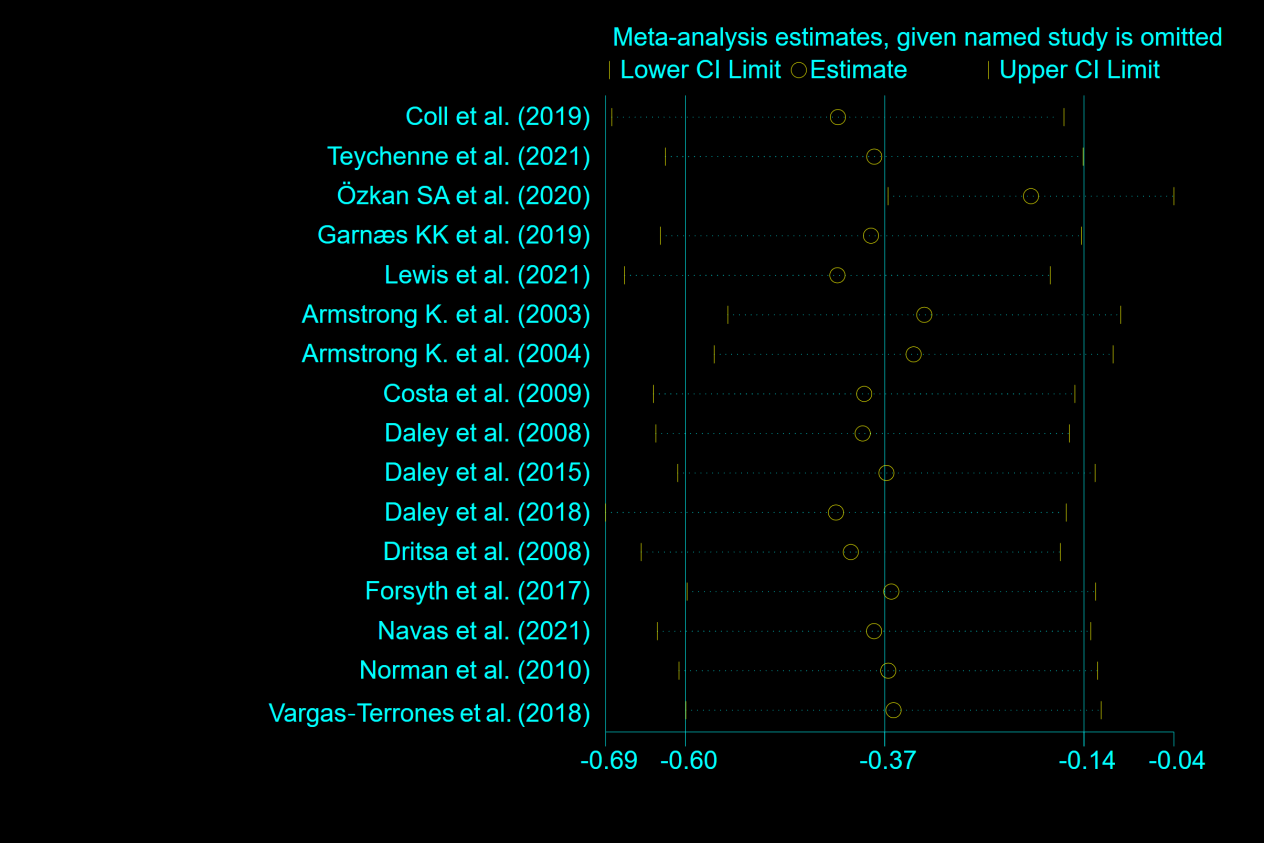


Figure 5.1 Sensitivity analysis plot for depression.


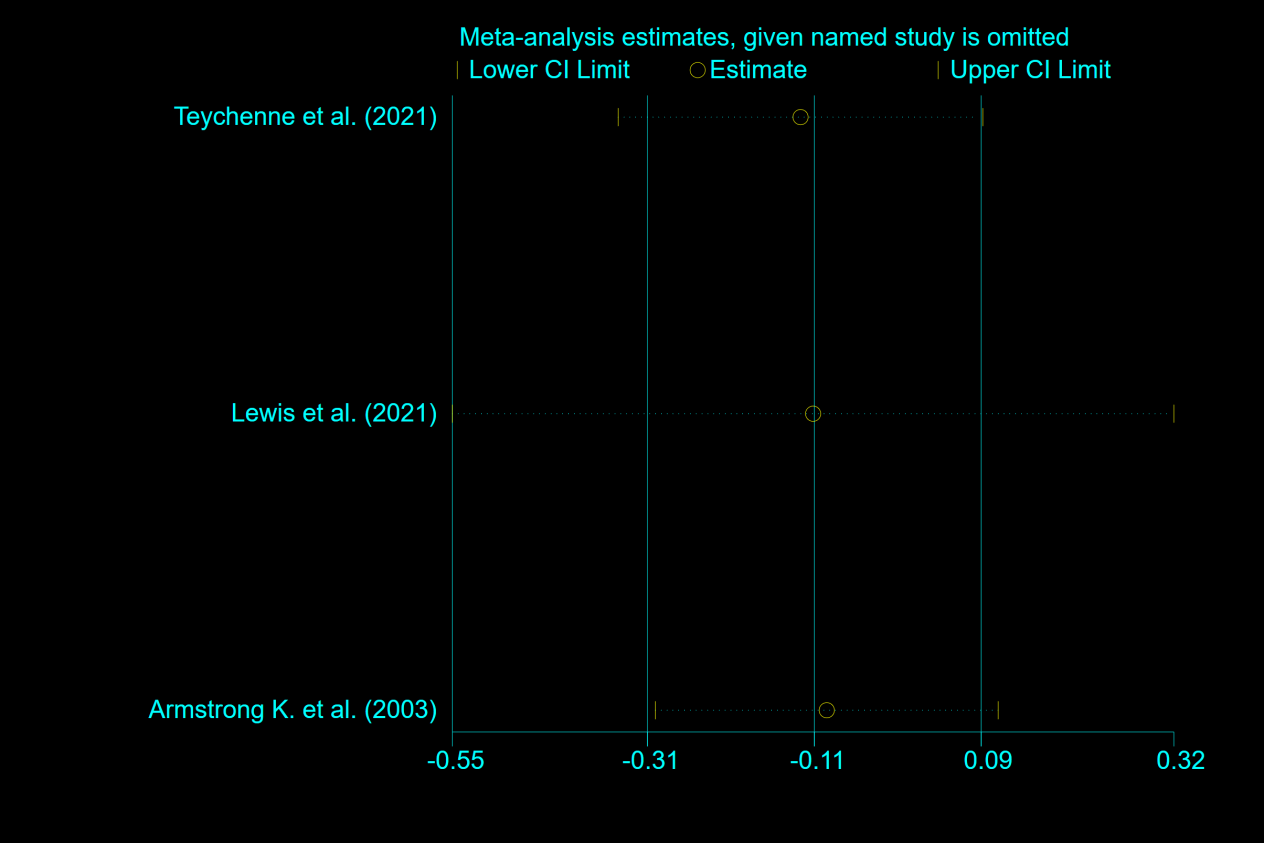


Figure 5.2 Sensitivity analysis plot for anxiety.


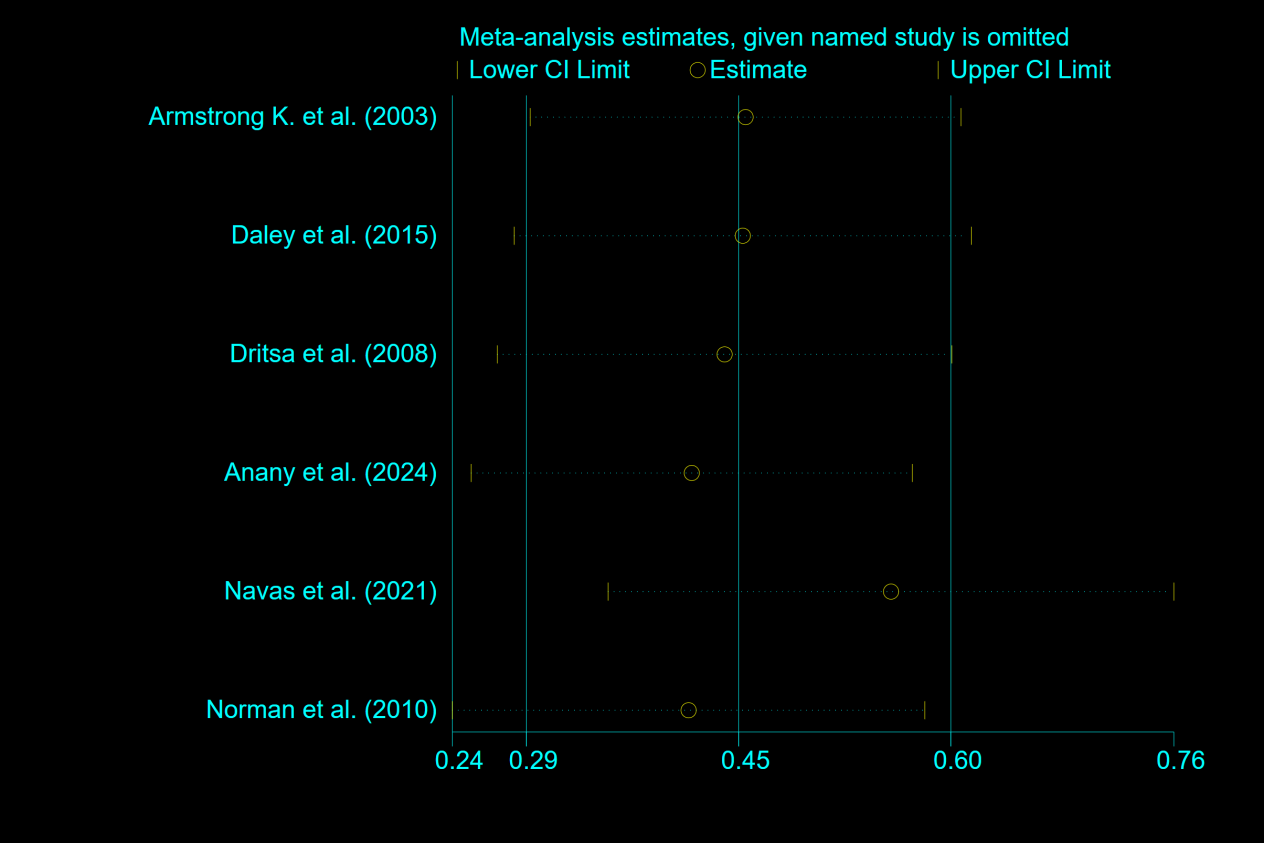


Figure 5.3 Sensitivity analysis plot for QoL.

# Supplementary 6: Publication bias


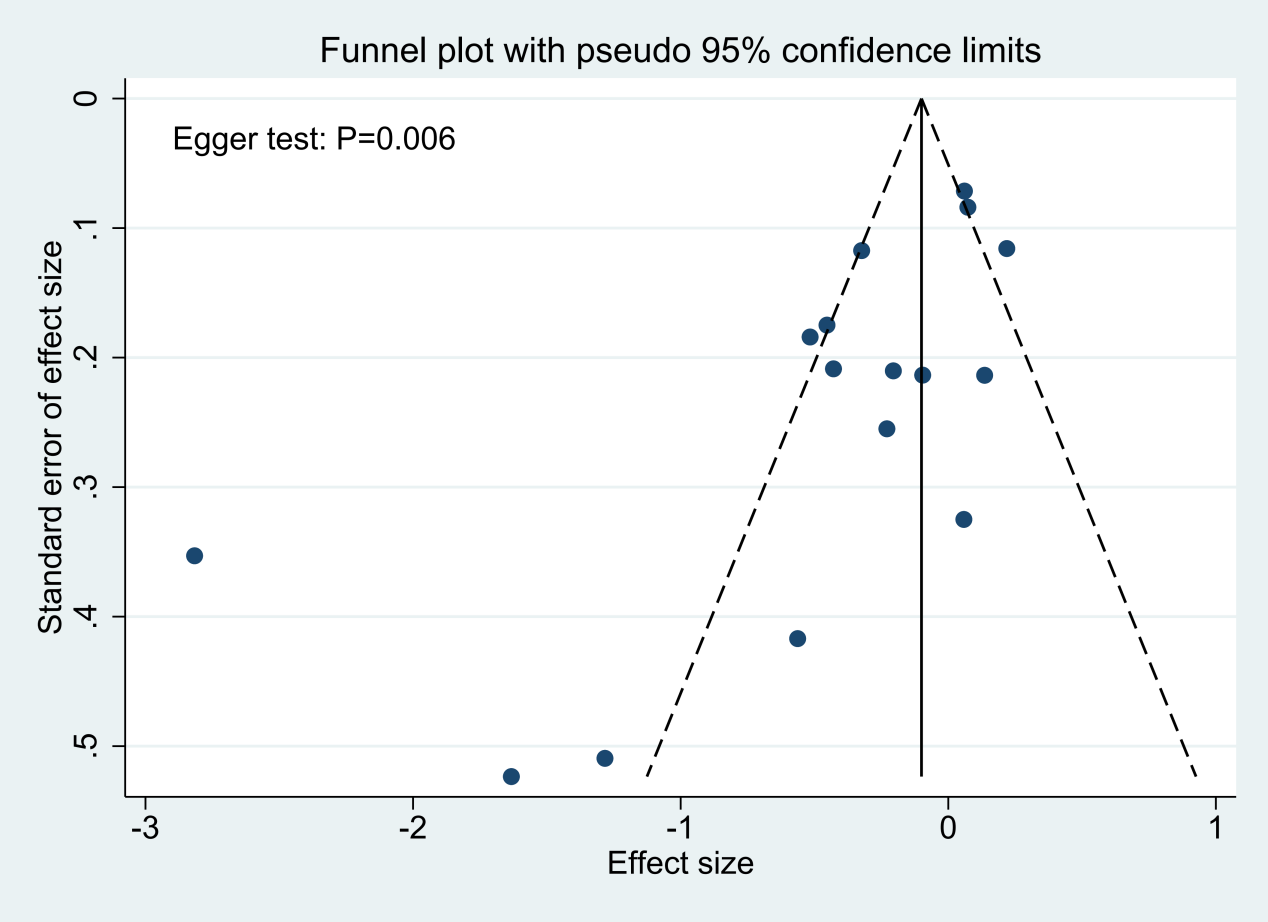


Figure 6.1 The funnel plot of depression. The result of Egger test showed the p=0.006.


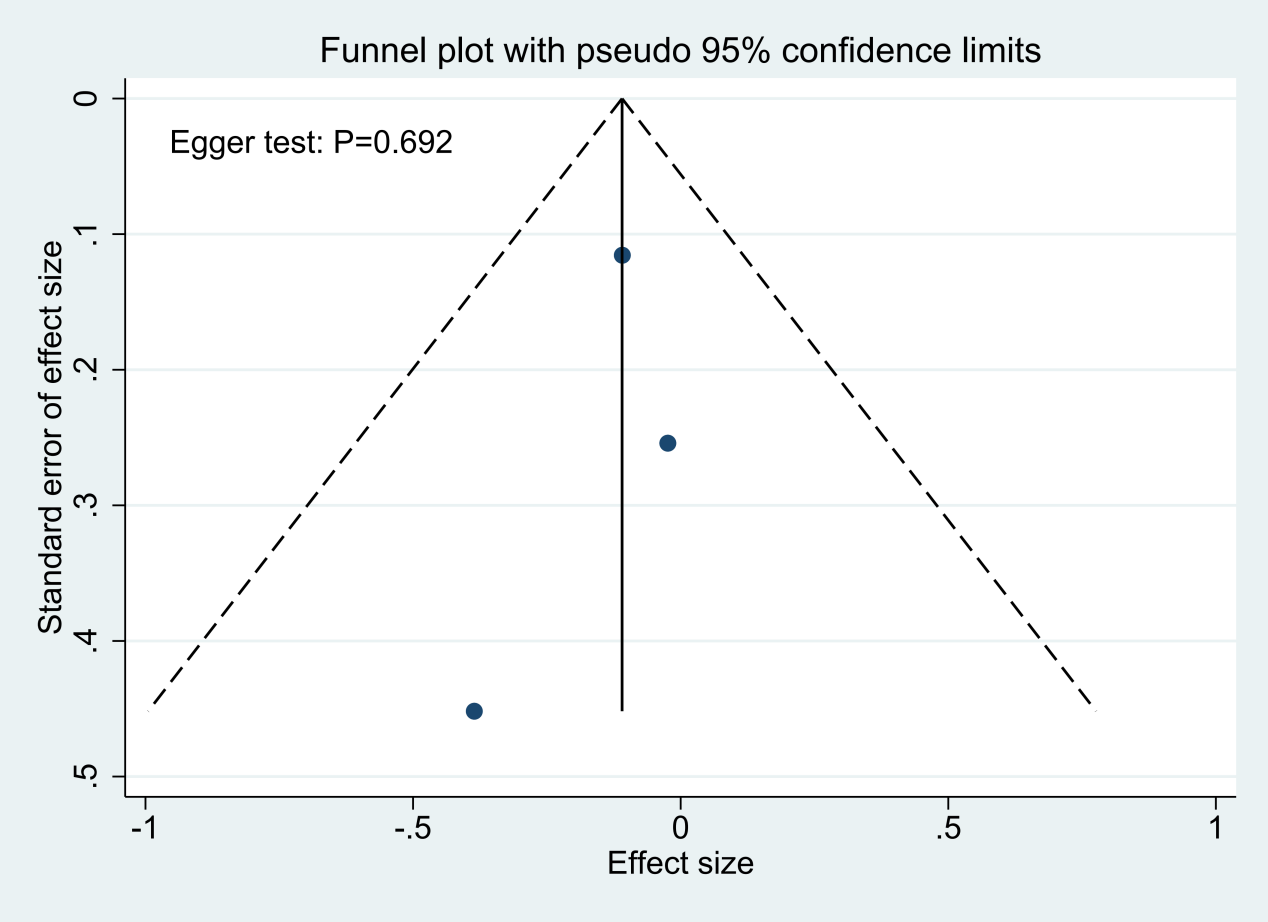


Figure 6.2 The funnel plot of anxiety. The result of Egger test showed the p=0.692.


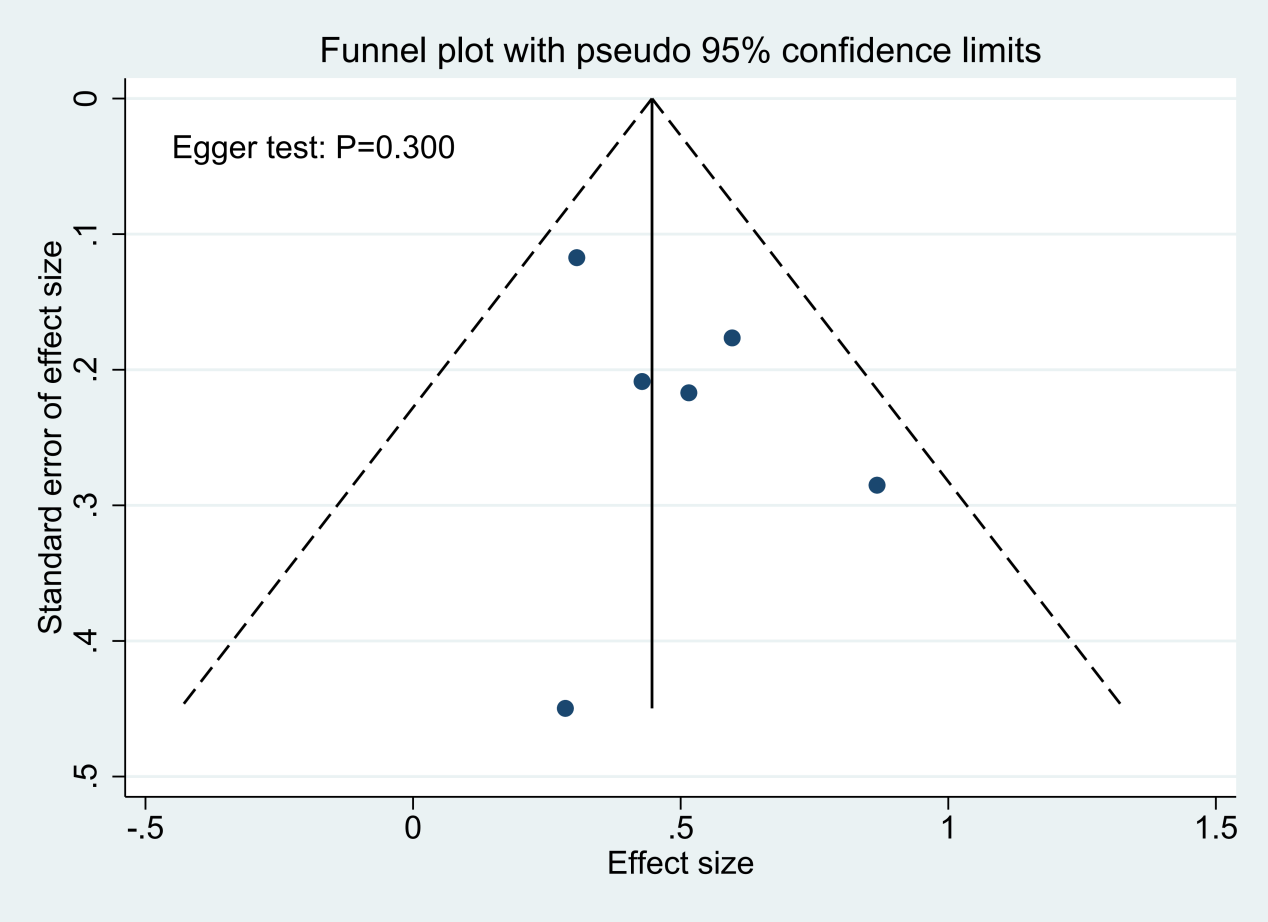


Figure 6.3 The funnel plot of QoL. The result of Egger test showed the p=0.300.
